# The ED/TEG Indicator for the Identification of Endocrine Disrupting or Toxic Effects on Endocrine Glands of Crop Protection Products Used in Organic and Conventional Agriculture in France

**DOI:** 10.3390/ijerph18073477

**Published:** 2021-03-27

**Authors:** Adèle Paul, Johan Spinosi, Mounia El Yamani, Anne Maitre, Barbara Charbotel

**Affiliations:** 1 UMRESTTE, UMR T 9405, Université Lyon 1, Université Gustave Eiffel—IFSTTAR, Domaine Rockefeller, 8 Avenue Rockefeller, 69008 Lyon, France; barbara.charbotel@univ-lyon1.fr; 2 Centre de Ressources en Pathologies Professionnelles et Environnementales, Centre Hospitalier Lyon Sud, Hospices Civils de Lyon, 165 chemin du Grand Revoyet, 69495 Pierre Bénite, France; 3 Santé Publique France, French National Public Health Agency, 12 rue du Val d'Osne, 94415 Saint-Maurice, France; johan.spinosi@santepubliquefrance.fr (J.S.); mounia.elyamani@santepubliquefrance.fr (M.E.Y.); 4 Occupational and Environmental Toxicology Laboratory, Biology and Pathology Institute, CHU Grenoble Alpes, 38700 La Tronche, France; anne.maitre@univ-grenoble-alpes.fr; 5 EPSP Team, TIMC IMAG Laboratory, UMR CNRS 5525, Université Grenoble Alpes, 621 Avenue Centrale, 38400 Saint Martin d’Hères, France

**Keywords:** pesticide, endocrine disruptors, occupational exposure, agriculture, endocrine organ

## Abstract

Studying the human health impacts of pesticides and their endocrine disruptor (ED) effects is a public health concern. The aim of this study is to identify phytopharmaceutical active substances (PAS) that are an ED or are toxic on endocrine glands (TEG), and to propose an ED/TEG effect indicator. Five international official databases were analyzed to identify the occurrence of health outcomes for 458 PAS. Health outcomes targeting seven endocrine systems were selected. For each substance, the level of evidence of the collected information and the number of outcomes were used to affect a level of concern about ED/TEG effects. Among the substances studied, 10% had a global ED/TEG effect classified as ‘high concern’, 55% as ‘medium concern’, 9% as ‘low concern’, and 26% as ‘unknown’. Ten of the high ED/TEG concern substances and 170 medium or low concern substances were licensed in 2018 in France. The outcomes were mainly on the reproductive organs, thyroid, and adrenal glands. Eight of the 41 biocontrol products studied were classified: 5 were ‘high’ or ‘medium concern’ and 3 had ‘unknown effect’. Although the proposed ED/TEG indicator is not an official classification, it can be used as an epidemiological tool for classifying the occupational and environmental risks of substances in retrospective population studies and be useful for occupational health physicians.

## 1. Introduction

The study of the human health impacts of pesticides is a public health action but is also essential for the understanding and control of this professional risk. Several crop protection products, including some that have been banned in Europe, are thought to be endocrine disruptors (ED) or to have toxic effects on the endocrine glands (TEG) [1]. ED act by disrupting the hormonal functions [2]. Thus, the activity of the endocrine glands can be altered. Through these effects, they are suspected of contributing to numerous chronic or developmental pathologies: infertility, obesity, thyroid disease, hormone-dependent cancers, immune disorders, etc. [3,4,5,6,7,8]. This study is important in the light of the significant 50 to 60% reduction in spermatozoid concentration in men, or the increased incidence of testicular cancer reported between 1973 and 2011 [2].

Although there was no consensus about the definition of ED, since the beginning of the 20th century, several organizations have made public their databases containing results from toxicology studies of crop protection products, particularly those with a potential endocrine mode of action. The European Union (EU) has initiated two programs for the identification of ED effects linked to exposure to crop protection products. These are the Endocrine Disruptor Strategy (EDS) and the Strategy for Identification of Endocrine Disruptors and Evaluation of their Cumulative Risks (European Food Safety Authority, EFSA) [9,10,11]. The European classification of carcinogenic and reprotoxic substances also provides information about the effects of these substances on endocrine organs [12]. The American Environmental Protection Agency (US-EPA) has implemented a screening program for ED effects of chemical substances, the Endocrine Disruptor Screening Program (EDSP), targeting specifically androgenic and estrogenic effects and those affecting the thyroid [13]. The World Health Organization (WHO) and the United Nations Organization for Food and Agriculture (FAO) have organized Joint Meetings on Pesticides Residues (JMPR) to establish the toxicological profiles of pesticides [14]. These databases are rich sources of diverse data that are often complex and even contradictory, with heterogeneous levels of evidence. The synthesis of information from these databases has enabled substances to be classified as having high, medium, and low ED/TEG concern by the official bodies. This classification could be useful to integrate and apply in population studies, especially as epidemiologists cannot rely on a formal regulatory classification as the EU regulation was quite recently defined.

Biocontrol products, defined as products using natural mechanisms in an integrated fight against unwanted pests and pathogens in culture could also be suspected of ED effects, particularly those containing chemical mediators, for example. The identification of phytopharmaceutical active substances (PAS) currently authorized for use in organic and conventional farming with potential ED/TEG effects would be useful to identify populations of workers exposed, and thus help to implement primary prevention strategies for exposure to these substances.

In France, the CIPA (Compilation des Index Phytosanitaires Acta, i.e., Compilation of Acta Phytosanitary Indexes) database contains information on all PAS that are listed in the Crop Protection Index from the Association for the Coordination of Farming Techniques (Acta) and have received product licenses since 1961 [15]. A toxicological module, the CIPATOX database, providing information on the health effects of PAS from regulatory and other official databases, was added in 2016. Eleven different health effects were registered, including ED, without any indication of the target organ, despite the fact that the targeted organs can be very diverse [16]. 

The objective of our study, CIPATOX-ED, was to identify the target organs for the ED/TEG effects of the PAS that have been licensed since 1961, and using data from the five official databases to construct an indicator reflecting the level of concern for the ED/TEG effects.

## 2. Materials and Methods

**Figure 1:**
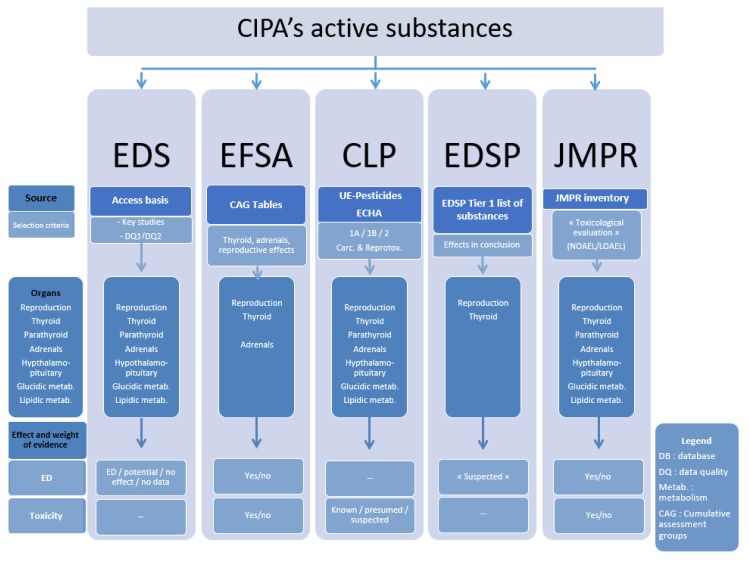
Summary of the endocrine disruptors, toxic effects, and target organs covered by the five databases used in this study.

We searched five databases, EDS, EFSA, EDSP, JMPR, and the Classification, Labelling and Packaging (CLP) list, which covered specific effects (disruptor or toxic) in different target organs as summarized in Figure 1. EDS is a European program developed in 2000 aimed at the identification by groups of experts of the ED effects of pesticides from data in the literature, according to the data quality [9]. The purpose of the Strategy for Identification of Endocrine Disruptors and Evaluation of their Cumulative Risks (EFSA) is to identify substances with similar toxicological properties for a specific organ or system (here thyroid, adrenals, and reproductive systems) [10,11]. The US-EPA’s EDSP, started in 1996, is a screening program, using validated testing systems and scientific data to identify androgenic, estrogenic, and thyroid ED effects of substances [13]. The JMPR (WHO-FAO) has been identifying the toxicological properties of SAPs from scientific toxicology studies since 1963 [14]. The CLP classification meets the European regulations on the classification, packaging, and labelling of hazardous chemicals. Among the hazards regulated by this CLP regulation are the carcinogenic or reprotoxic effects, classified according to their level of proof (1a: known to have an effect, 1b: presumed, 2: suspected) [12].

All ED and TEG effects were selected because of the lack of clear definitions when this work has been done, and the difficulty sometimes encountered in identifying the mode of action of toxic substances. Hence, this included all effects reported in the databases with a proven or suspected ED effect, based on study results, and also effects with an unknown or toxic mode of action. Toxic substances can lead to ED through an effect on endocrine synthesis, secretion, or the target organs. We then screened these effects to identify those that should be particularly assessed for their ED.

The target organs were selected using the definitions from the World Health Organization (WHO), and included the reproductive organs, the thyroid gland, the parathyroid glands, the adrenal glands, the hypothalamic–pituitary complex, the pancreatic islets and carbohydrate metabolism, and fatty tissue and lipid metabolism [17].

In each of the databases we registered the ED and toxic effects on the selected target organs for all the PAS that are in the CIPA database. In the EDS database we extracted data for the effects that had been studied in studies classified as ‘key studies’ or of good or sufficient quality by the database. In the EFSA database we extracted data for the effects that had been classed in a group for the evaluation of cumulative risk for reproductive organs or thyroid and adrenal glands. In the CLP we extracted data for level 1 and 2 carcinogenic or reproductive substances with definite or potential ED effects on an endocrine organ, as classified by the current provisional European Union definition at the time of this work. In this database, the PAS were classified as level 1A, 1B, or 2 carcinogenic or reproductive substances. In the EDSP database, we extracted data for substances with effects on adrenal and thyroid glands and estrogen judged to be pertinent by the database. In the JMPR database, we extracted data for substances cited in the conclusions of the toxicology profiles.

We synthesized the information extracted from the five databases and developed the ED/TEG indicator and used it to attribute an indicator for each substance and each target organ. The indicator has four levels to describe the ED concern: high, medium, low, and unknown effect. The indicator was developed to reflect the best available level of evidence for each effect for a given PAS. 

Only the EDS and CLP databases provided a classification with a level of evidence indicated for the ED effect in humans for the identified effects. In the EDS database, substances were classified as having a ‘proven’, namely ‘with evidence of ED’, or ‘potential’ ED effect, and in the CLP database, substances were classified as having ‘known (1A)’, ‘presumed (1B)’ or ‘suspected (2)’ carcinogenic or reprotoxic effects according to the provisional European Union definition. The development of the ED/TEG indicator was based on a ‘worst-case’ approach so that if one of the databases classified the ED effect as ‘proven’ for a given organ, the substance was classified as “high concern” for ED/TED effects, even if the other databases gave a lower certainty classification.

The analyses in the other three databases, EDSP, EFSA, and JMPR, were based on data obtained from in vivo animal studies or in vitro studies, and they did not attribute a level of evidence for the substances. Hence, we classified all the ED effects from these databases and those from the EDS and CLP databases that were not classified as proven as ‘potential ED/TEG’.

Since the ED effects of a substance for a given organ could have been studied in more than one of databases, we divided the substances with a ‘potential ED/TEG’ effect into ‘medium concern ED/TEG’ and ‘low concern ED/TEG’ based on the number of databases that analyzed the substance and identified the same effect (Table 1). These two categories are gathered into a ‘potential concern’ level of concern in our indicator.

PAS that had been assessed by the databases but had not been classified as high, medium, or low concern ED/TEG were considered as having ‘unknown ED effect’, based on the best available evidence. 

We established a global ED/TEG indicator representing the potential ED/TEG effect of a substance, irrespective of the target organ, so that the highest classification for a substance in any target organ was taken as the global ED/TEG indicator using a ‘worst-case’ approach. 

Information about marketing licenses was obtained from the EU pesticide database on the 5th February 2018. Information for 1990, 2000, and 2010 was obtained from the CIPA database [15,18].

The list of biocontrol products published by the French Ministry of Agriculture and Food, on the 16th of July 2018 in the French Journal Officiel was used as a reference to identify biocontrol products approved for organic farming in 2018 [19].

## 3. Results

A total of 458 PAS listed in the CIPA database were identified in the five databases (Table 2).

Using the ED/TEG indicator we developed for the 458 PAS identified, 44 (10%) were classified with ‘high concern’ ED or TEG, 254 (55%) as ‘medium concern’ ED or TEG, 40 (9%) as ‘low concern’ ED or TEG, and 120 (26%) as ‘unknown concern’ about ED or TEG. For the reproductive system we identified 32 (7%) substances classified as ‘high concern’ ED or TEG, 222 (48%) as ‘medium’, 49 (11%) as ‘low’, and 187 (41%) as ‘unknown concern’. For the thyroid there were 24 PAS (5%) as ‘high concern’ ED or TEG, 91 (20%) as ‘medium’, 50 (11%) as ‘low’, and 293 (64%) as ‘unknown concern’. For the adrenal glands there were no PAS classified as ‘high concern’ ED or TEG, 42 (9%) as ‘medium’, 40 (9%) as ‘low’, and 376 (82%) as ‘unknown concern’.

**Figure 2:**
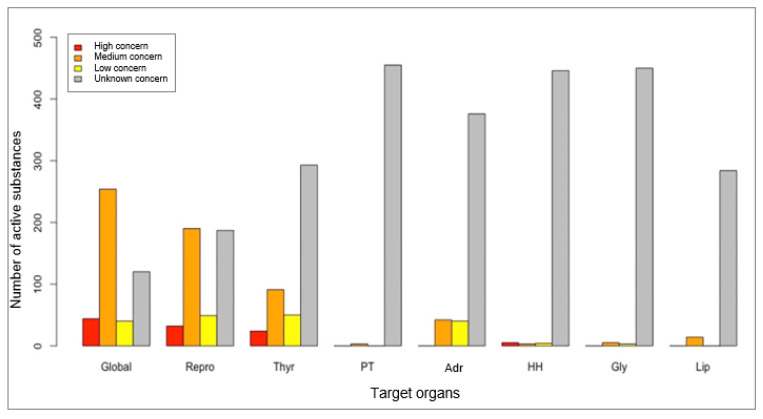
Number of phytopharmaceutical active substances by category of endocrine disruptor or target organ toxicity (ED/TEG) (Repro: reproduction organ, Thy: thyroid gland, PT: parathyroid glands, Adr: adrenal glands, HH: hypothalamic–pituitary complex, Gly: carbohydrate metabolism and pancreatic islets, Lip: lipid metabolism).

Only 14 (3%) of the PAS were found to have an ED or toxic effect on lipid metabolism, 12 (3%) on the hypothalamic–pituitary complex, 8 (2%) on carbohydrate metabolism and pancreatic islets, and 3 (1%) on the parathyroid glands (Figure 2).

On the 5th of February 2018, 334 PAS were licensed in France: 246 (74%) were included in our study and 88 (26%) were not found in any of the databases. Concerning the 246 PAS identified, 10 (4%) had a ‘high concern’ about ED/TEG effects, 151 (61%) had a ‘medium concern’, 19 (8%) had a ‘low concern’, and 66 (27%) had ‘unknown concern’ about ED or TEG effects.

Using our ED/TEG indicator, we found that at least 3% of the 334 PAS licensed in France in 2018 had an overall ‘high concern’ about ED/TEG effects, 45% had a ‘medium concern’, and 6% had a ‘low concern’. Thus 54% of the PAS licensed in France in 2018 have a medium or high overall ED or target organ concern. 

The numbers of PAS licensed in France in 2018 with a ‘high’ or ‘potential concern’ for ED/TEG effect are summarized by target organ (Table 3). The reproductive organs and the thyroid gland are the most targeted organs.

**Figure 3:**
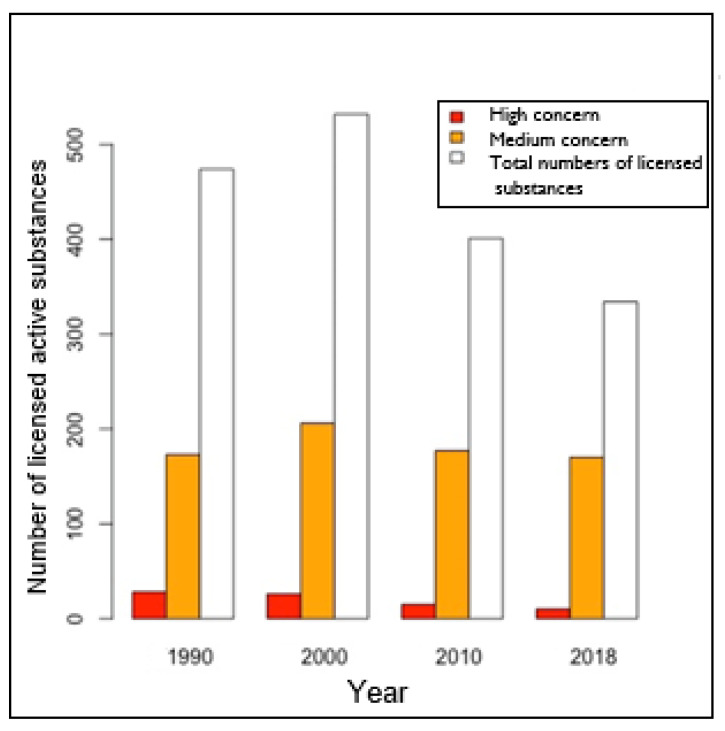
Evolution of the number of licensed phytopharmaceutical active substances in France classified as having a ‘high’ or ‘potential concern’ for endocrine disruptor or toxicity for an endocrine organ, using our ED/TEG indicator since 1990.

The number with a ‘high concern’ for ED/TEG has decreased from 1990 to 2018, while the number with a ‘potential concern’ for ED/TEG has remained stable, with a small increase in 2000, possibly linked to the increase in overall PAS licensed in that year (Figure 3). However, the percentage of PAS with a ‘high concern’ or ‘potential concern’ for ED/TEG has increased, with 42% in 1990, 44% in 2000, 48% in 2010, and 54% in 2018.

In France, 41 biocontrol products were approved in 2018: 8 (20%) were included in our study and 33 (80%) were not found in any of the databases. Using our ED/TEG indicator, one of these products had a global ‘high concern’ about ED/TEG, four had a global ‘medium concern’ ED/TEG and the remaining three were ‘unknown’ regarding their global effect ED/TEG concern (Table 4).

## 4. Discussion

The CIPATOX-ED project was initiated in 2018 to identify potential ED concerns in the general population from a public health point of view. We developed an ED/TEG indicator using data from five official international databases. This indicator was supposed to attribute to each PAS licensed in France a level of concern for ED effects, globally and for each target organ. We included all ED and organ toxicity effects, even those whose mode of action is unknown. Including only PAS with a known mode of action would not have been exhaustive, since the mode of action was rarely known, and would not have been appropriate for this public health project based on a precautionary approach.

The use of the five databases allowed us to assess a large number of PAS, but they each had their own specificities and could provide heterogeneous, even contradictory data, about the ED effects and their level of evidence. To use the level of evidence collected from the databases in the construction of the ED/TEG indicator, we attempted to limit their disparity by using the principle of the worse-case scenario, whereby if at least one of the databases reported a proven ED/TEG effect, this was retained, irrespective of the information in the other databases. 

Many substances were classified as ‘unknown effect’ based on our approach and the best available evidence. This should not be interpreted as meaning safe, but rather as an absence of conclusion showing an ED/TEG effect, either from documented scientific reasoning, lack of data, or absence of evaluation. Some of the databases concluded that there was no effect on specific target organs, for example the adrenal glands in the EFSA database, the thyroid gland in the EFSA and EDSP databases, and the reproductive organs in the EFSA database and the CLP list. These conclusions were added to the CIPATOX-ED database to enable ‘true negatives’ to be clearly identified with our current knowledge, but only for information purposed, and they are not described here. These conclusions of no effects can evolve as our knowledge increases.

From our experience with the five databases, it seems that the majority of the data collected concerns effects on the reproductive organs, thyroid, and adrenal glands, with fewer data for effects on carbohydrate and lipid metabolism, the parathyroid glands, or the hypothalamic–pituitary complex. This is not because the effects on these organs are less important, they have just been studied less, and therefore there are fewer data available. This may be explained by the fact that studies on reproduction and development are systematically included in product license dossiers and evaluations carried out by the JMPR. In general, the effects on the other organs are combined in short-term or long-term studies in which only the most protective toxicological reference values are considered. In addition, the number of effects identified for the thyroid or adrenal glands depends directly on the organs studied by EFSA via the cumulative risks evaluation groups. The high prevalence of overweight status and type II diabetes among farmers and their potential relationship with exposure to pesticides is a strong argument for further investigation into the effects of PAS on carbohydrate and lipid metabolism [20,21,22].

The aim of our project is to synthesize the conclusions from the five official databases in an indicator, the ED/TEG indicator, which can be used in population studies to provide information for the scale of the effects as a function of exposure to pesticides. Our results could also inform regulators about the substances recognized as being of particular concern and for which there is a priority to reduce or eliminate their use. They could also be useful to help occupational health physicians in the assessment between exposure to a PAS and a disease, or to set up clinical or biological monitoring adapted to a particular occupational environment. In addition, the ED/TEG indicator could be used to assess the environmental risk associated with these substances, as ED effects can occur even at very low dose exposure.

The importance of this primary prevention approach, which enables dangerous substances to be identified and substituted with safer products, is supported by the knowledge that it is difficult to ensure that farmers adhere to using individual and collective protection equipment [23].

It was not possible to study all PAS that were currently licensed in France as we included only those for which data existed in one of the five official databases. Some of these databases only contained data for substances that were suspected of having ED effects. This should have ensured that we covered substances with the highest risk of ED effects.

We could only study 8 of the 41 biocontrol substances approved in 2018, and 5 (12%) of these were classified as ‘high concern’ or ‘medium concern’ with the ED/TEG indicator, compared with 54% of the ‘conventional’ substances licensed in 2018. However, this comparison should be viewed with caution since 74% of the licensed ‘conventional’ substances were studied in CIPATOX-ED compared with 20% of the biocontrol substances approved in 2018. It could be important to note that there were only 41 biocontrol substances approved in 2018 in France, compared with 334 ‘conventional’ substances, which could limit any potential ‘cocktail’ effect and potential synergistic interactions between the products, and thus reduce the risk of any adverse effects in exposed users [17].

There has been a reduction in the number of licensed PAS since 1990, which could be due to the European directive published in 1991 that fixed the validity of PAS product licenses to 10 years [24]. This limitation could have led to certain products being withdrawn from the market because their toxicological or eco-toxicological properties did not meet the modern standards, it would be too expensive to perform the necessary evaluation studies, or there were insufficient economic interests to continue their commercialization. However, the percentage of substances classified as being globally of ‘high concern’ or ‘potential concern’ with the ED/TEG indicator among the licensed substances has increased since 1990. It would seem that despite more stringent toxicological or eco-toxicological principles, the specification requirements for marketing license application do not adequately and optimally take into account the ED/TEG effects of PAS. The European Council regulation No 1107/2009, published on the 21st of October 2009, concerning the placing of plant protection products on the market, states that PAS shall only be approved if it is ‘not considered to have endocrine disrupting properties that may cause adverse effect in humans’ [25]. In the absence of an official European definition of ED properties, the provisional definition, based on the CLP classification of substances, is probably not very efficient in identifying these properties [25]. 

Since the 20th of October 2018, the European Commission has adopted a definition for the determination of ED properties, which is aligned with the WHO 2002 definition, recognizing as ED all substances or mixture of substances that alter the function(s) of the endocrine system and thus induces “an adverse effect in an intact organism or its progeny, which is a change in the morphology, physiology, growth, development, reproduction or life span of an organism, system or (sub)population” [26]. It will be interesting to verify if this definition will improve the identification of ED and, thus, decrease the percentage of substances having high concern of potential ED effects among the licensed substances. This definition seems to be restrictive as it only retains substances with effects that have a known ED mode of action and does not take into account the secondary consequences of toxic effects [26]. During our study, this definition had not been adopted and the provisional definition, which took into account carcinogenic and reprotoxic substances, as used in the CLP classification, was still in force. Thus, although these results do not correspond to the definition in the CE 2018/605 regulation, they do highlight the legitimate need to reassess the ED effects of certain substances [26].

The CIPATOX-ED database, which can identify one or more target organ for each PAS, could also be used to assess the ‘cocktail’ effect from exposure of a target organ to several PAS or from several PAS with the same mode of action. Overall, CIPATOX-ED enabled the analyses of 458 PAS and showed that a considerable proportion of them potentially had ED/TEG effects, which could be due to a known endocrine mode of action or resulting from a toxic effect on an organ that could interfere with the synthesis, secretion, or elimination of the hormone involved. Some of these substances are licensed in France and other European countries. The methodological approach that we used aimed to overcome the difficulties related to this complex, polemic, topical subject by using several official databases from geographically different areas to propose an ED/TEG indicator based on the level of proof of the data extracted from the databases.

It will be interesting to follow this study with a study of all PAS in the CIPA database, including those that are currently licensed but also those that are banned, since these latter PAS could be persistent and therefore could still be responsible for exposure, or could have delayed, or even transgenerational, effects [27].

## 5. Conclusions

This study provides an identification of concerns about the ED/TEG effects of PAS used in France between 1961 and 2018. Since the regulations were still recent, epidemiologists did not have comprehensive databases at the time of this work. Comprehensive regulatory data would not be available quickly and probably not on old and prohibited PAS. The compilation of the databases carried out makes it possible to fill this gap and to improve the surveillance of the French population by estimating the prevalence of exposure to potentially ED/TEG PAS and the associated health effects, at a time when systems for monitoring the impact of ED on reproductive health are being studied on a European level [28]. This database could be used for the surveillance of exposed worker populations, but also for environmental exposures.

## Figures and Tables

**Table 1:** Classification of ‘potential ED/TEG’ into ‘medium concern’ and ‘low concern’ based on the number of databases in which they were classified.

Databases Identifying the Substance and Target Organ	Number of Databases Identifying a Potential Effect Required to Classify as a Medium Concern Endocrine Disruptor	Number of Databases Identifying a Potential Effect Required to Classify as a Low Concern Endocrine Disruptor
5	≥3	<3
4	≥3	<3
3	≥2	<2
2	≥1	
1	1	

**Table 2:** Number of phytopharmaceutical active substances (PAS) identified by database.

Database	Number of PAS Identified
EDS	141
EFSA	355
CLP	458
EDSP	45
JMPR	247
Total	458

**Table 3:** Number of phytopharmaceutical active substances (PAS) with a product license in 2018 in France identified with a high or potential concern for disruptor or toxic effect (ED/TEG) by target organ.

Target Organ	Licensed PAS with High or Potential Concern for ED/TEG Effect (*n*)
Reproductive organs	129
Thyroid gland	103
Parathyroid glands	2
Adrenal glands	56
Hypothalamic–pituitary complex	4
Pancreatic islets/carbohydrate metabolism	7
Lipid metabolism	10

**Table 4:** Phytopharmaceutical active substances for biocontrol licensed in 2018 and their levels of concern about endocrine disruption or toxicity for endocrine glands (ED/TEG)with target organ.

Biocontrol Substance	ED/TEG Concern	Target Organ(s)
Deltamethrin	High concern	Reproductive organs Hypothalamic-pituitary complex
Spinosad	Medium concern	Reproductive organs Thyroid gland Adrenal glands Carbohydrate metabolism
Pyrethrins	Medium concern	Thyroid gland (probable) Reproductive organs (possible)
Abamectin	Medium concern	Reproductive organs
Indole-3-butyric acid	Medium concern	Reproductive organs
Ferric phosphate	Unknown effect	
6-Benzyladenine	Unknown effect	
Gibberellins	Unknown effect	

## Data Availability

No new data were created or analyzed in this study. Data sharing is not applicable to this article.
